# Case of Lamellar Cataract, with Remarks

**Published:** 1857-09

**Authors:** E. Williams

**Affiliations:** Cincinnati


ART. VI.—*Case of Lamellar Cataract, with Remarks.*—By E. WILLIAMS, M.D., of Cincinnati.

THIS species of cataract, although long known to ophthalmologists by some of its striking characteristics, was first accurately diagnosed and described according to its *anatomical* peculiarities, by Edward Jäger, in his work, *Über Staar und Staaroperationen.* Prof. Arlt, of Prague, gives a short but very good description of it in his *Krankheiten des Auges,* under the name of *Stationäre Kernstaar jugendlicher Individuen.*

Mackenzie devotes merely a few lines to what he calls *central capsulo-lenticular cataract,* which I suppose to be, in fact, the disease under consideration. But the first elaborate account, and one which will ever do honor to his name, is that given by Dr. A. v. Graefe, of Berlin, in the second part of the first volume of the *Archiv für Ophthalmologie,* page 235, issued in April, 1855, of which he is one of the editors. In the second part of the second volume of the same journal for 1856, is a report of some interesting cases of the same affection by Dr. D. E. Müller, of Oldenburg. Dr. Jäger gave it the very appropriate name of *Schichstaar,* or *Lamellar Cataract,* and this is the appellation which is now applied to it by all German ophthalmologists.

As to its frequency, there is much discrepancy. Jäger, at the time his book was published, had only observed this variety of opacity of the lens in 4 cases in some 910 individuals affected with cataract; whereas Graefe reckons it the most common form of disease of the lenticular system in children. Dr. Müller saw some 70 or more instances in the space of fifteen months. Arlt had observed it in 16 to 20 persons, between the ages of 8 and 40 years. I myself had an opportunity of examining several cases while in attendance upon the clinic of Dr. Graefe, in Berlin, during the winter of '54 and '55. Here, in Cincinnati, but two examples have come under my notice, one of which I will hereafter detail; the other was shown me by my friend, Dr. Krause.

Early life seems to be the period at which the disease, by far, most frequently develops itself. Whether ever congenital or not is an unsettled point; that it is sometimes hereditary is probable, from the fact stated by Dr. Müller, that he has seen it in three sisters, whose mother was very *near-sighted* from her earliest childhood, a fact which was very probably due to the same state of the lenses in her eyes. It occurs very likely most often before the seventh year, but being at first only slightly marked, it escapes notice till the child is put to school. Graefe says, he has never seen it before the fourth year, because, probably previous to that time, the child not being required to exercise very close sight, it goes unnoticed.

One thing is quite certain, and that is, that medical advice is usually sought in such cases for supposed myopia, or deficiency of sight, the cataract not having been detected by the parents. Such patients are not unfrequently referred to the physician by the optician for advice, in consequence of his inability to find any glass that suits the case. Whether congenital, or occurring at a very tender age, the disease usually makes pretty rapid progress for some months or a year or so, and then remains stationary for an indefinite time, and most commonly, as would seem, for the remainder of life. Prof. Arlt has observed several such patients for from 5 to 10 years, without being able to detect the slightest change either in the color or volume of the opacity.

In one patient, who said he had been *nearsighted* since his earliest school days, and who, for the first time, found it necessary to change the concave glasses he had always worn, when he was 40 years old, there was good reason to believe the disease had remained stationary all that time. It has been not unfrequently seen, however, to begin anew after a certain period of quiescence, and go on to complete opacity of the entire lens.

Schichtenstaar or Lamellar Cataract is a circumscribed opacity of a thin lamina of the lens, the peripheric cortical portion of the organ and the nucleus remaining perfectly transparent.

The particulars of the following case, which is in reality a *type one*, will supersede the necessity of a lengthy description of the diagnostic peculiarities of this interesting and till recently but little understood variety of cataract.

H. S., æt. 17, a farmer by trade, of a lymphatic temperament and rather feeble physical development, consulted me some weeks ago in regard to an infirmity of vision. The father states that the patient has been *nearsighted* and had a *peculiar expression* about his eyes, since his *earliest childhood*, and his sight has always been defective. Since recovering from an attack of scarlatina, about nine years ago, his vision has been worse than before. A careful examination of the case led to the discovery of the following interesting points.

When not fixed upon an object his eyes wander or oscillate slightly, as is seen in mild nystagmus. Pupils rather small but active, and in each is seen a diffused bluish-white opacity, nearly uniform throughout, which has its seat evidently in the crystalline body.

Near the superior margin of the left, when it is moderately dilated by shading the eye with the hand, is a distinct white patch, about the size of a small pin's head, which extends down upon the diffused bluish-white opacity, towards the middle of the pupil.

In order to a more satisfactory inspection, I dilated the pupils with atropia, when the nature of the obstruction to vision was strikingly displayed.

Occupying the middle of each pupil, but on a plane a little behind it, is a circumscribed spot or disk, from 2 to 2½ lines in diameter, terminating abruptly at its border, and surrounded by a peripherical zone of the lens that is perfectly transparent.

The degree of saturation is very nearly the same in both eyes, with the exception of the white spot superposed upon the disk in the left one. Viewed in a darkened room, by the aid of a bright lamp and the ophthalmoscope, there is seen a state of things which I shall describe as faithfully as possible, protesting all the time, however, that to *see such a case once* is far more satisfactory than to peruse a thousand descriptions. Examined by perpendicular light, that is, when the light reflected from the ophthalmoscope falls perpendicularly to the plane of the pupil, in the *left eye*, the opacity presents a reddish-brown color, the red predominating in the centre, where it is more translucent than towards its circumference. The translucency is greater in the central part of the disk, under such circumstances, not because there is really less opacity there, but because of the perpendicular direction of the incident rays favoring their transmission in larger quantities than towards the circumference, where, falling more obliquely, they are more numerously reflected. Examined in a *side view*, either by changing the position of the instrument or directing the patient to turn the eye to one side, a very different appearance is presented. The whole circular surface assumes now a uniform light-brown color, except at the circumference, where it is bordered by a narrow strip or ring, more deeply saturated with white. From the outer edge of this whitish border a number of short radiating lines spring out, which give it a stellated appearance.

When a strong double convex lens is used, these radii are seen to be very numerous, and the edge, which appears nearly smooth to the naked eye, has a ragged or rather fine radiating aspect. External to this there is a transparent zone, corresponding to the periphery of the lens.

In the right eye the disk is also semi-transparent, but *less so just in the centre* than a little more externally. It is also more deeply saturated around its circumference, as in the other eye, and between this whitish border and the centre is a zone, more translucent than either the circumference or the centre. From the inferior and internal edge a narrow ribbon-like stripe branches off in the direction of a radius, and, after a course of about one-quarter of a line, dilates into a sort of button, from which emanate two diverging branches, each of which terminates by an enlarged extremity, a little short of the edge of the lens, one being somewhat larger than the other. By an oblique view, I can see beyond the ends of each of these eccentric emanations. At other points of the circumference, numerous small radiating lines are visible, some five or six of which project beyond the rest. The vitreous humor is perfectly transparent, and the retinal vessels are easily seen, but only in the peripheric portions of the illuminated background of the eyes, the optic papilla and central parts of the retina being obscured or eclipsed by the opaque disk in the lens. In order not to attach undue importance to the opthalmoscope, in the examination of such a case, it is proper to mention that the peculiarities of the cataracts are so salient as to be recognized by the unassisted eye, when the lamp is held very close to the patient's face. But the use of the instrument makes them much more striking, gives a far better idea of the relative translucency of the different parts, and enables one to discover the fine radii that go off from the edges of the disks, far more distinctly than with the naked eye. For this latter object the magnifying lens is indispensable, only the large branches above described, and some five or six of the numerous radii, being visible without it.

As to the vision of the patient, he sees badly at all distances, and appears very myopic when made to examine a minute object. In attempting to read, he holds the book about four inches from the end of his nose, and even then makes out common-sized print with difficulty. His sight is not perceptibly benefited by any sort of glasses, for he has made repeated trials of all kinds; nor has it changed for the last six or eight years. As soon as I dilate the pupils, however, he can see a great deal better in the distance, and is able to read with tolerable ease at ten or twelve inches. This is plainly due to the fact that, when the pupils are of the ordinary size, they are smaller than the opaque disks, and the images on the retina are of course then very little illuminated. Besides, the irregular refraction effected by the opaque parts of the lens, causes additional confusion of sight. When dilated, a large zone of transparent lens is uncovered, and a much larger amount of light admitted.

I made the diagnosis of *lamellar cataract*, that is, a central circumscribed opacity of a lamella of the crystalline lens, that lies between the anterior cortical substance and the nucleus, both of which parts are clear, at least in one eye. The opthalmoscopic inspection enables me to detect some difference in the state of the two lenses. In the left, it is a simple *Schichten Cataract*, as defined above. But in the right, there is the appearance of two central opacities, occupying two distinct lamellæ, which are separated by a thin portion or layer of clear lenticular substance.

In the last-mentioned eye there may be also some opacity of the centre of the nucleus, but I cannot certainly make that diagnosis. To make the matter clear to the comprehension of every reader, it may be said, that if we proceed along the axis of the lens, we have first the anterior capsule and cortical substance quite healthy, next an opaque lamella, then a transparent one, then another opaque, and finally the nucleus clear, or, as it is probably in the right eye, somewhat turbid. So far as I know, only four or five cases have been reported, where it was pretty certain that there were two or more opaque laminæ, separated by an intervening clear portion, and none where there was at the same time an opaque nucleus.

As respects treatment, the patient had, fortunately for his sight, resisted the importunities of flocks of *itinerant eye doctors,* who, to use the forcible language of an eccentric medical friend, have "*gone a whoring* with the word *oculist.*" Such impertinent, pestiferous vagabonds, now infest our medical Israel in swarms, such as "neither our fathers, nor our fathers' fathers have seen, since the day that they were upon the earth!"

O! for a professional Moses, that might find favor with the Lord, and successfully entreat Him to smother out the vile frogs, and flies, and lice, and locusts, that have been showered down upon us, in retribution for the woful and unpardonable neglect of the scientific profession, in acquainting themselves with the beautiful science of ophthalmology!

But to return, from these slimy stinking *bull frogs*, that bellow themselves into temporary notoriety from the columns of nearly all our newspapers, and filch money from the pockets of an ever-itching-to-be-humbugged people, to our patient, who has been blessed with mother wit enough to turn away with suspicion from the stupid or knavish quacks, who have insisted upon *depressing his cataracts*, than which nothing could be much more certainly fatal to his vision. I advised him to submit to no operation, but keep the pupils dilated with atropia as long as the disease remains stationary, which will probably be for the remainder of his life. I was induced to recommend this course to him in consequence of the improvement of sight produced by dilating the pupils, and the unusual ease with which he adapts the eyes to vision at different distances, notwithstanding the dilatation; the harmlessness of the remedy; but above all the serious contingencies that so often follow, even the best-chosen and most skilfully-performed operation, in consequence of rapid and great swelling of the lens, in these cases. But two operations are at all admissible in this variety of cataract, and they are,—careful laceration of the capsule through the cornea, or artificial pupil, as practised by Dr. Graefe. In a subsequent number, I propose to devote another article to this subject, in which I shall have an opportunity of speaking more at length in regard to the period *when* operative interference is justifiable, and the method best adapted to the nature of the disease.

